# The B & B approach: Ball-milling conjugation of dextran with phenylboronic acid (PBA)-functionalized BODIPY

**DOI:** 10.3762/bjoc.16.188

**Published:** 2020-09-11

**Authors:** Patrizia Andreozzi, Lorenza Tamberi, Elisamaria Tasca, Gina Elena Giacomazzo, Marta Martinez, Mirko Severi, Marco Marradi, Stefano Cicchi, Sergio Moya, Giacomo Biagiotti, Barbara Richichi

**Affiliations:** 1 Department of Chemistry ‘Ugo Schiff’, University of Florence, Via della Lastruccia 3/13, 50019 Sesto Fiorentino, FI, Italy; 2 Soft Matter Nanotechnology Group, CIC biomaGUNE, Paseo Miramón 182 C, 20014 San Sebastián, Guipúzcoa, Spain; 3 Chemistry Department, University “La Sapienza”, P.le Aldo Moro 5, 00185 Rome, Italy; 4 NanoBioMedical Centre, Adam Mickiewicz University in Poznań, Wszechnicy Piastowskiej 3, 61-614 Poznań, Poland

**Keywords:** ball milling, boronic ester, dextran, bodipy, nanoparticles

## Abstract

Mechanochemistry is an emerging and reliable alternative to conventional solution (batch) synthesis of complex molecules under green and solvent-free conditions. In this regard, we report here on the conjugation of a dextran polysaccharide with a fluorescent probe, a phenylboronic acid (PBA)-functionalized boron dipyrromethene (BODIPY) applying the ball milling approach. The ball milling formation of boron esters between PBA BODIPY and dextran proved to be more efficient in terms of reaction time, amount of reactants, and labelling degree compared to the corresponding solution-based synthetic route. PBA-BODIPY dextran assembles into nanoparticles of around 200 nm by hydrophobic interactions. The resulting PBA-BODIPY dextran nanoparticles retain an apolar interior as proved by pyrene fluorescence, suitable for the encapsulation of hydrophobic drugs with high biocompatibility while remaining fluorescent.

## Introduction

In the last few decades, mechanochemistry has gathered a great deal of attention and a lot of efforts have been focused on its use in organic synthesis, catalysis, biotransformation reactions, and even in nanomaterials preparation [1–5]. Indeed, solid-state mechanochemical methodologies are a viable alternative to traditional syntheses in solution [4] for the preparation of complex molecules, either under solvent-free conditions, named ‘neat grinding’, or in nearly solvent-free conditions, e.g., liquid-assisted grinding (LAG), slurries, and homogenous solutions. Although there are compounds that can only be achieved by conventional solution-based methods, mechanochemistry offers some important advantages over conventional bulk synthesis. These advantages have become more evident with the development of atomized ball milling technologies, which allow for a better reproducibility and control of reaction parameters over traditional mortar or pestle [4,6]. Mechanochemical syntheses are not just a means for cleaner, safer, higher yielding, and more sustainable chemical transformations, but also lead to the synthesis of compounds otherwise elusive with conventional solution synthesis [7]. Moreover, mechanochemical milling protocols allow chemical transformations using poorly soluble compounds. In this regards, mechanochemical milling is less affected by solvent conditions as bulk chemistry and thus it makes possible the synthesis of moisture sensitive compounds, without requiring inert atmosphere conditions necessary for their synthesis in solution. In this framework, mechanochemical approaches have been recently investigated for the straightforward synthesis of a wide range of biomolecules: amino acids [8], peptides [9], glycosides [10], nucleosides/nucleotides [11], and lipids [12]. Also, protein-based nano-bio-conjugates [13] have been prepared by ball milling, retaining the native properties of the proteins after mechanochemical synthesis.

Boronic acids and their related esters are relevant synthetic building blocks widely employed as cross-coupling reagents [14] as well as protecting groups for polyols and diamines [15–16]. Moreover, the reversible covalent interaction of boronic acids with specifically oriented *cis*-1,2 and 1,3-diols has been successfully exploited in many applications including the selective sensing of saccharides, the separation and purification of glycoproteins, and the immobilization of antibodies in microarrays [17–21]. However, the synthesis of boronic esters is sometimes problematic, as it often requires a fine tuning of the reaction conditions (i.e., high temperatures, inert atmosphere, control of pH of the media), and the continuous removal of water (i.e., Soxhlet extractor, Dean-Stark trap, molecular sieves) to drive the reaction to completion [22–23]. Finally, depending on the substrates, these reactions often proceed with low conversions and/or yields even if a large excess of reactants is employed. These processes are sometimes time-consuming and require additional purification steps [24–25]. In this context, ball milling procedures can be considered a valid alternative approach for the straightforward synthesis of boronic esters. Indeed, ball milling has been successfully exploited for the synthesis of small molecules [4] as well as macrocycles [5,22–23] conjugated with boronic esters and this methodology proved to be by far superior over solution-based methods.

**Figure 1:**
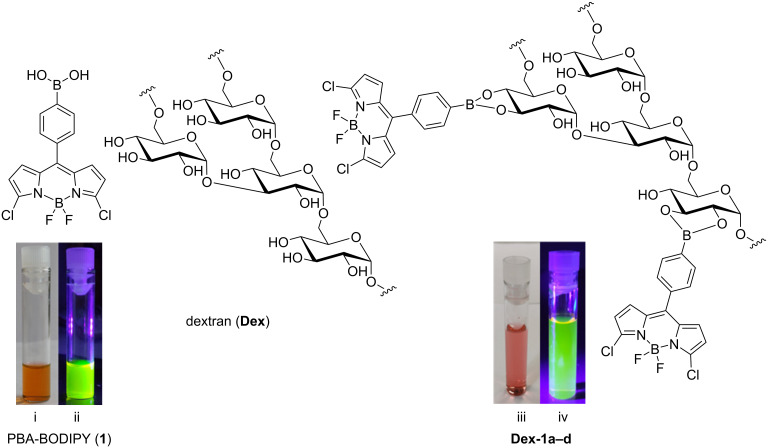
Structure of PBA-BODIPY (**1**) and schematic representation of dextran (**Dex**) and PBA-BODIPY conjugated dextran **Dex-1a**–**d**. The structure of **Dex-1a/d** has not been experimentally defined in this article but it has been depicted as boronate ester as previously described [27–28]. Solution of PBA-BODIPY (**1**, at 1.0 mg/mL in H_2_O): i) under white light and ii) under UV light (λ_ex_ = 364 nm). Solution of **Dex-1b** (at 1.0 mg/mL in H_2_O): iii) under white light and iv) under UV light (λ_ex_ = 364 nm).

In this framework, we decided to exploit the mechanochemical approach (the B & B, ball milling for boronic acid conjugation) as bioconjugation strategy for the labeling of biocompatible carbohydrates. Fluorescent labeling is of key importance to follow up the fate of molecules and (nano)materials inside cells and in the human body. In this regard, we recently reported on the straightforward synthesis of probe **1** (Figure 1) where a phenylboronic acid (PBA) moiety was introduced at the *meso* position of a BODIPY core. We demonstrated the compound’s ability to bind the oligosaccharide chain of the Fc domain of monoclonal antibodies, through the formation of a boronate ester [26].

In this work we investigated the mechanochemical preparation of fluorescently labeled dextran exploiting the formation of boronate esters with the phenylboronic acid moiety of PBA-BODIPY (**1**). Dextran (**Dex**, Figure 1) 9,000–11,000 Da was chosen due to a series of characteristics generally associated to dextran polysaccharides: they are highly soluble in water, biodegradable, biocompatible, commercially available, and cheap. Furthermore, the presence of hydroxy groups allows for an easy chemical functionalization with complementary functional groups. Indeed, dextrans and their derivatives have been widely used in biomedical applications, especially as drug and gene carriers, both in hydrogels and in nanoparticulate formulations [29–32]. In this regard, the incorporation of hydrophobic moieties in the branched structure of dextrans induces self-assembly into nanoparticles, depending on the degree of substitution. For example, reaction of vicinal diols of dextran with hydrophobic PBA generates boronate esters, which form nanoparticles in water and are capable to host the anticancer drug doxorubicin [27].

In this report, the advantages of the milling process, such as short reaction time, reactant economy, higher degree of functionalization, and solvent-free conditions, compared to solution-based routes are discussed. The dextran mechanochemically conjugated to PBA-BODIPY (**Dex-1b**, Figure 1) formed nanoparticles through self-assembly retaining the fluorescent properties of BODIPY and the biocompatibility of dextran. The BODIPY dextran nanoparticles were characterized regarding their size, morphology, polarity, and toxicity in vitro. We demonstrate the feasibility of mechanochemistry for boronic ester formation [23] to a glycan polymer as a route of conjugation with small molecules, in a green and scalable process.

## Results and Discussion

**Scheme 1:**
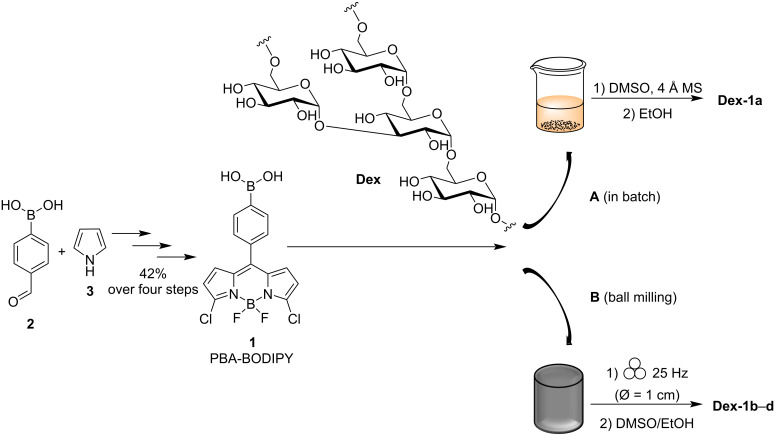
Schematic representation of dextran/PBA-BODIPY bioconjugations in: A. conventional solution-based conditions to prepare the **Dex-1a** conjugate (using roughly 1 equiv of **1**). B. Ball milling conditions to prepare conjugates **Dex-1b** (using roughly 1 equiv of **1**), **Dex-1c** (using roughly 0.5 equiv of **1**), and **Dex-1d** (using roughly 0.1 equiv of **1**).

The boron dipyrromethene dye **1** bearing a phenylboronic acid moiety (PBA) at the *meso* position of the BODIPY core (Scheme 1) was prepared in four synthetic steps starting from 4-formylbenzeneboronic acid (**2**) and pyrrole (**3**), by following a straightforward synthetic strategy recently reported by some of us [26].

Then, in order to assess the efficacy of the solid-state milling approach in the formation of boronate esters between the PBA moiety of **1** and the vicinal diols of dextran (**Dex**), both mechanochemical and conventional solution-based (in batch) protocols were investigated. In solution, dextran functionalization was carried out following a modified protocol recently reported on the conjugation of PBA to dextran [27]. This protocol was based on a conventional solution-based approach [27], where a significant excess of PBA (more than 60-fold excess of PBA to dextran, in a glucose/PBA 1:1 molar ratio reaction) was employed. However, the resulting dextran functionalization was low (1.6 PBA units out of 60 glucose units). In our studies we used PBA-BODIPY (**1**), a dye which proved to show excellent optical properties for biofunctionalization [26] and that displays a PBA moiety conjugated to a BODIPY core. The mechanochemical synthesis avoids the use of a large excess of **1** in the reaction mixture. Accordingly, reactions following either mechanochemical or solution-based protocols were carried out using a roughly equimolar amount of **Dex** and **1**, in a glucose units/**1** molar ratio of 60:1 (assuming an average molecular weight for **Dex** of 10 kDa).

For the labeling in solution, **Dex** was dissolved in dry dimethyl sulfoxide (DMSO) and PBA-BODIPY (**1**) was added in roughly equimolar amounts (Scheme 1, route A). The reaction was carried out by stirring in the presence of 4 Å molecular sieves for 6 h at room temperature [27]. The conjugate (**Dex-1a**, Scheme 1, route A) was precipitated from cooled ethanol (EtOH) and filtered. Then, the pink solid was suspended in ethanol and the suspension was placed in an ultrasonic bath (59 kHz, 5 min) and filtered. This protocol was repeated and then, the solid was washed over the filter with ethanol and dried under vacuum. The DMSO/EtOH mixture and the two serial ethanol washings were kept separated for further analysis (vide infra).

The mechanochemical reaction was performed by grinding **Dex** and **1** (in roughly equimolar amounts) at 25 Hz for 90 minutes, in a 10 mL mixer mills using one stainless-steel ball (∅ = 1.0 cm). The product, a red powder (**Dex**-**1b**, Scheme 1, route B), was easily recovered from the jar and solubilized in DMSO. Then, the conjugate (**Dex-1b**, Scheme 1, route B) was precipitated from cooled ethanol (EtOH) and filtered. The resulting solid was suspended in ethanol and placed in an ultrasonic bath (59 kHz, 5 min) and filtered. This protocol was repeated, and the precipitate washed over the filter with ethanol and dried under vacuum. The DMSO/EtOH mixture and the two ethanol washings were kept separated for further analysis (vide infra).

**Figure 2:**
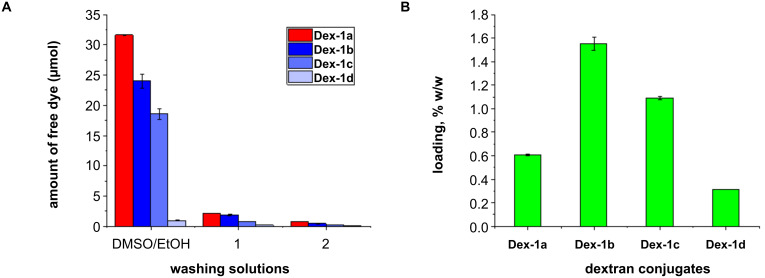
A) Amount of recovered PBA-BODIPY (**1**, i.e., nonreacted **1**) in the mixtures DMSO/EtOH and in the serial ethanol washing solutions related to the reactions in solution (red bars) and the ball mill (blue bars), specifically: **Dex-1a** (reaction in DMSO with roughly 1 equiv of **1**), **Dex-1b** (ball milling with roughly 1 equiv of **1**), **Dex-1c** (ball milling with roughly 0.5 equiv of **1**), and **Dex-1d** (ball milling with roughly 0.1 equiv of **1**). B) PBA-BODIPY (**1**) loading in the conjugates **Dex-1a** (reaction in DMSO solution with roughly 1 equiv of **1**), **Dex-1b** (ball milling with roughly 1 equiv of **1**), **Dex-1c** (ball milling with roughly 0.5 equiv of **1**), and **Dex-1d** (ball milling with roughly 0.1 equiv of **1**) expressed as % w/w on the modified dextrans.

In order to evaluate the outcome of the two approaches in terms of dextran labeling with BODIPY, firstly, the amount of the recovered PBA-BODIPY (**1**, i.e., nonreacted **1**) in each ethanol washing solution was quantified by UV–vis spectroscopy (λ_max_ = 508–511 nm). The DMSO/ethanol mixtures and ethanol solutions were dried under vacuum, the recovered dye was solubilized in DMSO, and the UV–vis spectra were recorded. The amount of recovered PBA-BODIPY (**1**) was estimated (Figure 2A), using the molar extinction coefficient of PBA-BODIPY which was evaluated in DMSO (see Supporting Information File 1, Figure S1).

Figure 2A shows the amount of unreacted **1** recovered either from the serial ethanol washing solutions or from the DMSO/EtOH mixtures related to the solution-based (red bars, Figure 2A) and ball mill (blue bars, Figure 2A) route. The largest amount of free PBA-BODIPY (**1**, i.e., nonreacted **1**) was recovered from the DMSO/EtOH washings for both solution (**Dex-1a**) and ball milling (**Dex-1b**) reactions. In addition, the difference between the two approaches was clear: the free PBA-BODIPY (**1**) recovered (i.e., nonreacted **1**) in the conventional approach was higher compared to the ball billing approach (Figure 2A). The yield of both, solution-based and ball mill conjugations was monitored by measuring the absorption of the conjugates (λ_max_ = 508–511 nm) and the relative amount of conjugated PBA-BODIPY (**1**) was calculated (Figure 2B) according to the molar extinction coefficient (see Supporting Information File 1, Figure S1). Considering that dextran is a polydisperse polymer the functionalization was reported as weight ratio percent in the labeled polymer [26]. As shown in Figure 2B, a 1.6% w/w of **1** in the **Dex-1b** conjugate (milled reaction) compared to a 0.61% w/w of **1** in the **Dex-1a** conjugate (conventional solution reaction) was obtained. The loading of **1** in both conjugates was also confirmed by inductively coupled plasma atomic emission spectroscopy (ICP-AES) analysis which showed in **Dex-1b** a boron content roughly eight times higher compared to **Dex-1a** (0.98 and 0.12 mg/g, respectively).

These results demonstrated that the ball-milling approach provides a higher degree of functionalization, with a loading almost three times higher in the ball mill compared to conventional solution synthesis. In addition the ball-milling approach allowed to obtain similar results in terms of loading as previously reported by Levkin [27], but, notably, using a more than 60 times lower amount of the PBA-BODIPY bearing moiety avoiding unnecessary excess of the dye (roughly 1 equiv of PBA-BODIPY vs 60 equiv of PBA).

To get further inside in the yield of the ball-milling reaction, we investigated to what extent the amount of PBA-BODIPY (**1**) in the reaction mixture affected the loading of the fluorescent probe. For this purpose, reactions were repeated using, respectively roughly 0.5 (**Dex-1c**) and 0.1 (**Dex-1d**) equivalents of **1** compared to **Dex**. Therefore, mechanochemical reactions were carried out using a glucose units/**1** molar ratio of 120:1 and 600:1. The conjugations yield was determined by measuring the absorption of the conjugates (λ_max_ = 508–511 nm, Figure 2B and Supporting Information File 1, Figures S3 and S4), and calculating the relative amount of conjugated PBA-BODIPY (**1**) [26]. Of note, a good linearity was observed: the 1.6% w/w of functionalization in the **Dex-1b** conjugate (related to the reaction with 1 equiv of **1**), decreased up to 1.1% w/w in the conjugate **Dex-1c** (related to the reaction with 0.5 equiv of **1**) and up to 0.3% w/w in the conjugate **Dex-1d** (related to the reaction with 0.1 equiv of **1**). As before, this linear trend of the loading of **1** in all the conjugates (**Dex-1b**–**d**) was also confirmed by ICP-AES analysis (0.98, 0.6 and 0.09 mg/g, respectively).

**Figure 3:**
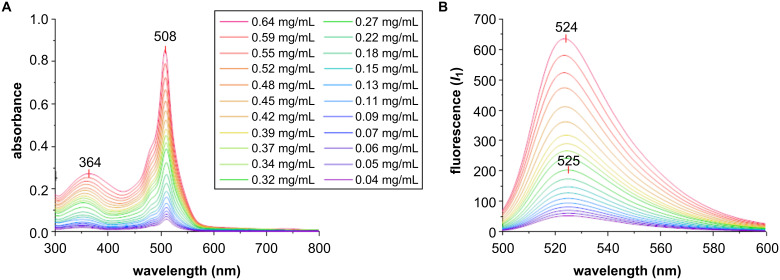
A) UV–vis absorption and B) fluorescence emission spectra (λ_exc_ = 380 nm) of the BODIPY-dextran conjugate **Dex-1b** solution in water.

Then, the **Dex-1b** conjugate was further characterized by means of UV–vis spectroscopy, Fourier-transform infrared spectroscopy (FTIR), dynamic light scattering (DLS), and transmission electron microscopy (TEM) analysis (see Supporting Information File 1). Concerning the chemical structure of the conjugate, the data reported in the literature were sometime discordant [27–28,33–34]. The structure of conjugates **Dex**-**1a**–**d** was not experimentally defined here and, in Figure 1, it has been depicted as boronate ester as previously described [27–28] for analogous systems to provide explicitness to the figure. Then, the spectroscopic properties of the PBA-BODIPY-dextran conjugate **Dex-1b** were studied in water. The UV–vis spectra of **Dex-1b** reported in Figure 3A shows the same absorption pattern of the PBA-BODIPY (**1**, see Supporting Information File 1) [26].

The main absorption band corresponding to the transition S_0_ → S_1_, is observed at λ_abs_ = 508 nm. The shoulder at shorter wavelength (λ_abs_ = 364 nm) is typical for a symmetric dye. Normalized fluorescence spectra of conjugate **Dex-1b** were recorded at two different excitation wavelengths (λ_exc_ = 380 and 460 nm) and they are shown in Figure 3B and Figure S2 (Supporting Information File 1). The maximum emission bands were at λ_em_ = 524 nm (λ_exc_ = 380 nm) and λ_em_ = 523 nm (λ_exc_ = 460 nm, see Supporting Information File 1, Figure S2). Then, a Stokes shift of 16 nm could be appreciated in agreement with data previously reported for PBA-BODIPY (**1**) [26,35].

**Figure 4:**
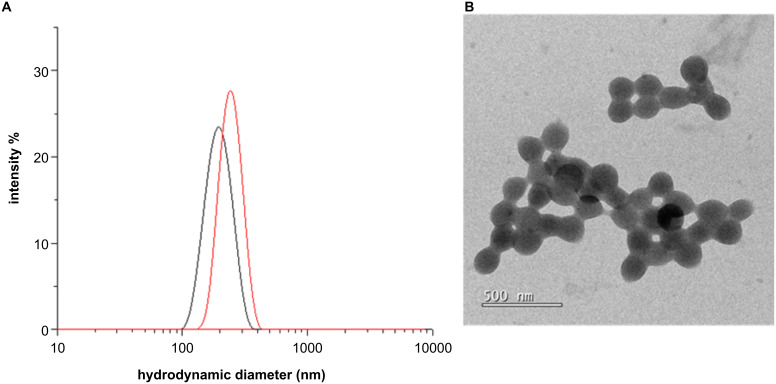
A) Hydrodynamic diameter of (nm) conjugate **Dex-1b** (at 1 mg/mL in H_2_O, black curve) and PBS (red curve), respectively. B) TEM image of conjugate **Dex-1b** in H_2_O.

Dynamic light scattering (DLS) measurements were performed for the conjugate **Dex-1b** in water and in phosphate-buffered saline (PBS) solution (Figure 4A, see Experimental). Nonfunctionalized dextran showed very low scattering and poor autocorrelation functions as there is limited association among dextran chains and single chains have limited scattering (data not shown). Instead, for the conjugate **Dex-1b** the scattering was high with a well-confirmed autocorrelation function (Figure S5 in Supporting Information File 1). Thus, the DLS data confirmed that the conjugate **Dex-1b** forms aggregates or nanoparticles as previously reported [27]. The size (hydrodynamic diameter) of the nanoparticles in H_2_O was 198 ± 42 nm (Figure 4A, black). The measurements were performed also in phosphate-buffered saline (PBS) solution to evaluate a potential additional aggregation in cell medium. The aggregates were stable in solution and did not precipitate over time. The size of the nanoparticles dispersed in PBS was slightly larger compared to those in H_2_O, the hydrodynamic diameter was 246 ± 44 nm (Figure 4A, red). Moreover, TEM images (Figure 4B) showed that the nanoparticles of conjugate **Dex-1b** were quite polydisperse with a size comparable to the one obtained by DLS.

**Figure 5:**
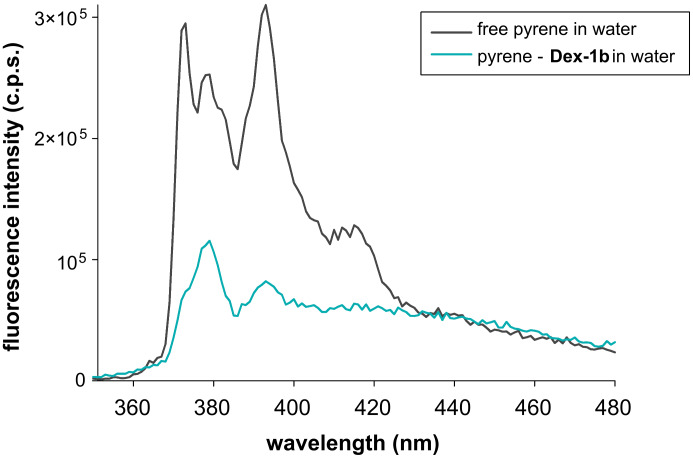
Fluorescence emission spectra of pyrene (4.4 × 10^−8^ M) in water and in a water solution in the presence of conjugate **Dex-1b**.

As discussed in the introduction, it has already been shown that PBA conjugation leads to the hydrophobic association of dextran [32], and being **1** larger than PBA and more hydrophobic it can reasonably generate hydrophobic environments in the dextran chain, which results in the association of the conjugate **Dex-1b** in aggregates or nanoparticles. Moreover, it is known that dye conjugation to a polymer even in small percentages can generate a large change in the environment of the chains affecting conformation and chain organization [36]. To assess the influence of hydrophobicity on the formation of assemblies of the conjugate **Dex-1b** a simple experiment with pyrene was conducted. It is well known that the fluorescence spectrum of pyrene is very sensitive to the polarity of the environment [37]. Indeed, the ratio (*I*_1_/*I*_3_) between the first (*I*_1_ = 372 nm) and the third bands (*I*_3_ = 383 nm) in the emission spectra of pyrene could be used to estimate the polarity of the environment of the dye in an empirical way. The ratio of the two bands in an environment of unknown polarity can be compared with the values of this ratio in solvents of known polarity. The spectrum of pyrene dissolved in a water dispersion of the conjugate **Dex-1b** and the one of pyrene in water as reference are shown in Figure 5.

The spectra of pyrene in a solution with nonconjugated dextran was very similar to that of water (data not shown). We observed that there was a significant change in the relative intensity of the first and third bands in between the spectra in water and in those with the conjugate **Dex-1b** solution [37]. The *I*_1_/*I*_3_ of pyrene was 1.1 in water, while in the conjugated dextran solution it was 1.5, a value which is comparable with solvents like diethyl malonate, dioxane, or dimethoxyethane, all organic solvents highly miscible with water, but practically apolar. We assumed that pyrene, with a limited water solubility was completely located inside the dextran assemblies. If a significant fraction of pyrene was located in the water phase the spectra would resemble more the spectra in water or in nonconjugate dextran. The pyrene spectra proved an apolar environment for the assemblies of the conjugate **Dex-1b** that could be indeed due to the covalent attachment of **1**, which in turn triggers association of dextran chains. It may also be that a dense array of dextran molecules could have the polarity characteristics observed. It is difficult to give a definitive answer, but the experiments demonstrated that the environment inside the dextran nanoparticles favored the solubilization of organic molecules like pyrene. This characteristic of the nanoparticles could bring applications in drug delivery since they are stable in aqueous media, without further functionalization, but can trap apolar molecules in the interior, as was shown in [27] for nanoparticles formed from dextran boronated in bulk. These nanoparticles formed by hydrophobic interactions were capable of solubilizing doxorubicin and liberated the drug intracellularly through hydrolysis of the boronic esters.

Indeed, the formation of assemblies or nanoparticles of the conjugate **Dex-1b** capable of solubilizing organic molecules and retaining water stability could be of interest for drug delivery, considering the exceptional biocompatibility of the dextran. We also performed an MTT assay of the conjugate **Dex-1b** to evaluate its toxicity and we could observe basically no effect on cell proliferation in the A549 cell line (see Figure S6 in Supporting Information File 1), which remained above of 60% after 24 hours of incubation over the whole concentration range tested.

In general, the outcomes of using PBA-BODIPY (**1**) instead of PBA alone did not seem to largely change the characteristics of the dextran nanoparticles produced in terms of size and biocompatibility. However, the presence of BODIPY had the advantage of rendering the nanoparticles fluorescent, and this property could be used to trace the conjugate in a biological milieu.

## Conclusion

We have shown that ball milling technologies could be effectively applied for the conjugation of PBA-BODIPY (**1**) to dextran through the formation of boronate esters between the phenylboronic acid group of PBA-BODIPY and the hydroxy groups of glucose monomers in dextran. The synthesis required less amounts of BODIPY and was more effective (higher yield) than the conventional solution synthesis. The fluorescent BODIPY dextran conjugate spontaneously assembled in nanoparticles with sizes around 200 nm. The assemblies had an apolar interior that makes them appealing for encapsulation of organic molecules. Since the assemblies retained the superior biocompatibility of dextran we envisage their use in drug delivery, also profiting from the fluorescence properties of the nanoparticles that could be used to trace them in biological environments.

## Experimental

### Materials and methods

All reagents, whose synthesis is not described, were commercially available and were used without any further purification, if not specified otherwise. Ball milling was carried out with a Retch MM 400 mixer mill. Filtration was carried out using a glass vacuum filter with 0.2 µm sartorius. UV–vis spectra were recorded on a Varian Cary 4000 UV–vis spectrophotometer using a 1 cm cell or a BioTeK Synergy H4 microplate reader. Fluorescence spectra were registered on a Jasco FP750 spectrofluorometer using a 1 cm cell. NMR spectra were recorded on an Inova 400 instrument. Fourier transform infra red absorption spectroscopy (FTIR) was carried out with a Shimadzu IRAffinity-1S spectrometer. Potassium bromide (KBr) pellets were prepared using 100 mg of potassium bromide and 1.0 mg of sample. The spectra were recorded under inert atmosphere and a pure potassium bromide pellet was used as background. Inductively coupled plasma atomic emission spectroscopy (ICP-AES) was used to determine the boron (B) concentrations and was performed in triplicate using a Varian 720-ES Inductively Coupled Plasma Atomic Emission Spectrometer (ICP-AES). An accurately weighted amount of each sample was treated with a microwave-assisted digestion (CEM MARS Xpress) using 1 mL of suprapure HNO_3_ obtained by sub-boiling distillation and 1 mL of suprapure H_2_O_2_. Each sample was thus diluted to 10 mL with Ultrapure water (UHQ), spiked with 0.5 ppm of Ge used as an internal standard, and analyzed. Calibration standards were prepared by gravimetric serial dilution from commercial stock standard solutions of B at 1000 mg L^−1^. The wavelength used for B determination was 249.772 nm whereas for Ge the line at 209.426 nm was used. The operating conditions were optimized to obtain maximum signal intensity, and between each sample, a rinse solution constituted of 2% v/v HNO_3_ was used to avoid memory effects. Dynamic light scattering (DLS) measurements were carried out with a ζ-Sizer Malvern Instrument in the backscattering mode. All studies were performed at a 173° scattering angle with temperature controlled at 25 °C in 1 mL polystyrene cuvettes. **Dex-1b** was characterized in terms of size. Short time measurements were carried out for a total of 15 min, with three consecutive measurements for each sample. Transmission electron microscopy images were aquired by a JEOL JEM 1010 microscope, operating at an acceleration voltage of 120 kV. A fresh solution of **Dex-1b** was prepared at a concentration of 10 mg/mL in ultra-HPLC water, and sonicated for 15 s at room temperature. A short spin of 30 seconds at 10 krpm was applied in order to remove the big aggregates and dust. The stock solution was diluted 1:2 in ultra-HPLC water, and 1 μL of the solution was deposited on the top of the TEM grid and left dried at room temperature. Steady state fluorescence measurements were performed on a Fluorolog^®^-3 spectrofluorometer (Horiba-Jobin Yvon) using 1.0 × 1.0 cm quartz cuvette from Hellma. λ_exc_ = 335 nm, acquisition range: 350–500 nm, slits: 2/2 nm, [pyrene] = 4.4 × 10^−8^ M in water. Pyrene fluorescence was measured using a stock solution of pyrene prepared in methanol due to its poor solubility in water (with a concentration of 4.45 × 10^−6^ M). Ten µL of this solution were dissolved in 1.0 mL of water to obtain a final concentration of 4.4 × 10^−8^ M.

### Cell culture

Human lung adenocarcinoma (A549) cells were cultured in RPMI 1640 medium supplemented with 10% (v/v) fetal bovine serum (FBS) and 1% (v/v) antibiotic solution (100 units/mL penicillin, 100 mg/mL streptomycin, P/S). The cells were maintained at 37 °C, with 5% CO_2_ in a humidified chamber.

### Cell viability MTT assay

Cell mitochondrial activity was tested using the 3-(4,5-dimethylthiazol-2-yl)-2,5-diphenyltetrazolium bromide (MTT) assay, which is based on the mitochondrial conversion of the tetrazolium salt into a formazan dye with absorption characteristics in the visible region. Cells were incubated with **Dex-1b** at different concentrations. After 24 h the cells were washed and 135 μL fresh medium with 15 μL of MTT (at 5 mg/mL in PBS) were added to each well. Culture plates were then incubated at 37 °C for 2 h, after which the medium-containing MTT was discarded and formazan crystals were dissolved in 150 µL DMSO. The absorbance at 550 nm (with automatic discount of reference wavelength 630 nm) of the resulting solution was measured in a 96-well spectrophotometer microplate reader. The percentage of cell mitochondrial activity was determined by the following formula: (Absorbance of treated cells/ Absorbance of control cells) × 100 (%).

### Synthesis of 3,5-dichloro-4,4-difluoro-8-(4-boronophenyl)-4-bora-3a,4a-diaza-*s*-indacene (PBA-BODIPY, **1**)

PBA-BODIPY (**1**) was prepared following the synthetic strategy reported in ref [26]. NMR and HRMS data were published in ref [26]. FTIR data are shown in Figure S12 of Supporting Information File 1

### Synthesis of BODIPY-Dextran conjugates

#### Dex-1a

In a dried sealed tube under a nitrogen atmosphere were added 4 Å molecular sieves (100 mg), 400 mg of 9–11 kDa dextran (at least 0.044 mmol), 17 mg of PBA-BODIPY (**1**, 0.044 mmol), and 6.6 mL of dry dimethyl sulfoxide. The mixture was stirred for 6 h, then the product was precipitated in cold ethanol (50 mL), and filtered over a 0.2 µm nylon membrane. The powder was recovered and dispersed (6.8 mg/mL) in ethanol and sonicated for 5 min at 59 kHz. The dispersion was filtered over a 0.2 µm nylon membrane recovering both solid and solution. This protocol was repeated and then the solid was washed over the filter with ethanol (2 × 30 mL) and dried under vacuum to obtain 250 mg of **Dex-1a** as orange solid. FTIR data are shown in Figure S7 of Supporting Information File 1. ICP-AES boron content: 0.12 mg/g.

#### Dex-1b

A 10 mL stainless-steel mixing mill equipped with a 1 cm diameter ss ball was charged with 400 mg of 9–11 kDa dextran (at least 0.044 mmol), 17 mg of PBA-BODIPY (**1**, 0.044 mmol), and milled for 90 min at 25 Hz. The powder was recovered, dissolved in DMSO (6.6 mL) and the product precipitated from cold ethanol (50 mL) and filtered over a 0.2 µm nylon membrane. The powder was recovered and dispersed (6.8 mg/mL) in ethanol and sonicated for 5 min at 59 kHz. The dispersion was filtered over a 0.2 µm nylon membrane recovering both solid and solution. The last protocol was repeated and then the solid was washed over the filter with ethanol (2 × 30 mL) and dried under vacuum to obtain 380 mg of **Dex-1b** as dark orange-solid. Boron content measured by ICP-AES 0.98 mg/g. FTIR data are given in Figure S8 of Supporting Information File 1.

#### Dex-1c

A 10 mL stainless-steel mixing mill equipped with a 1 cm diameter ss ball was charged with 400 mg of 9–11 kDa dextran (at least 0.044 mmol), 8.5 mg of PBA-BODIPY (**1**, 0.022 mmol), and milled for 90 min at 25 Hz. The powder was recovered, dissolved in DMSO (6.6 mL) and the product precipitated from cold ethanol (50 mL) and filtered over a 0.2 µm nylon membrane. The powder was recovered and dispersed (6.8 mg/mL) in ethanol and sonicated for 5 min at 59 kHz. The dispersion was filtered over a 0.2 µm nylon membrane recovering both solid and solution. The last protocol was repeated and then the solid was washed over the filter with ethanol (2 × 30 mL) and dried under vacuum to obtain 336 mg of **Dex-1c** as orange-solid. FTIR data are given in Figure S9 of Supporting Information File 1. ICP-AES boron content 0.61 mg/g.

#### Dex-1d

A 10 mL stainless-steel mixing mill equipped with a 1 cm diameter ss ball was charged with 400 mg of 9–11 kDa Dextran (at least 0.044 mmol), 1.7 mg of PBA-BODIPY (**1**, 0.0044 mmol), and milled for 90 min at 25 Hz. The powder was recovered, dissolved in DMSO (6.6 mL) and the product was precipitated from cold ethanol (50 mL) and filtered over a 0.2 µm nylon membrane. The powder was recovered and dispersed (6.8 mg/mL) in ethanol and sonicated for 5 min at 59 kHz. The dispersion was filtered over a 0.2 µm nylon membrane recovering both solid and solution. The last protocol was repeated and then, the solid was washed over the filter with ethanol (2 × 30 mL) and dried under vacuum to obtain 360 mg of **Dex-1d** as light orange-solid FTIR data are given in Figure S10 of Supporting Information File 1. ICP-AES boron content 0.09 mg/g.

## Supporting Information

File 1 Methods for UV–vis and fluorescence measurements, UV–vis and fluorescence spectra of **Dex-1b, Dex-1c**, and **1** in DMSO, FTIR spectra of **PBA-BODIPY 1**, dextran and **Dex-1a**–**d**; MTT assay and correlation coefficient of **Dex-1b**.
