# Supplementary material for: The B & B approach: Ball-milling conjugation of dextran with phenylboronic acid (PBA)-functionalized BODIPY
Source: Beilstein J Org Chem. 2020 Sep 11;16:2272–81. doi: 10.3762/bjoc.16.188 (PMC7492696; doi:10.3762/bjoc.16.188)
Supplement: File 1 — Methods for UV–vis and fluorescence measurements, UV–vis and fluorescence spectra of Dex-1b, Dex-1c, and 1 in DMSO, FTIR spectra of PBA-BODIPY 1, dextran and Dex-1a–d; MTT assay and correlation coefficient of Dex-1b. [file Beilstein_J_Org_Chem-16-2272-s001.pdf]

BEILSTEIN JOURNAL OF ORGANIC CHEMISTRY

Supporting Information

for

The B & B approach: Ball-milling conjugation of dextran with phenylboronic acid (PBA)-functionalized BODIPY

Patrizia Andreozzi, Lorenza Tamberi, Elisamaria Tasca, Gina Elena Giacomazzo, Marta Martinez, Mirko Severi, Marco Marradi, Stefano Cicchi, Sergio Moya, Giacomo Biagiotti and Barbara Richichi

Beilstein J. Org. Chem. **2020,** *16,* 2272–2281. doi:10.3762/bjoc.16.188

Methods for UV–vis and fluorescence measurements, UV–vis and fluorescence spectra of Dex-1b, Dex-1c, and 1 in DMSO, FTIR spectra of PBA-BODIPY 1, dextran and Dex-1a–d; MTT assay and correlation coefficient of Dex-1b

License and Terms: This is a supporting information file under the terms of the Creative Commons Attribution License (https://creativecommons.org/licenses/by/4.0). Please note that the reuse, redistribution and reproduction in particular requires that the authors and source are credited.
The license is subject to the *Beilstein Journal of Organic Chemistry* terms and conditions: (https://www.beilstein-journals.org/bjoc)

Table of contents

**UV–vis and fluorescence measurements**	S2
**Figure S1**	S2
**Figure S2**	S3
**Figure S3**	S3
**Figure S4**	S4
**Figure S5**	S4
**Figure S6**	S5
**Figure S7**	S5
**Figure S8**	S6
**Figure S9**	S6
**Figure S10**	S7
**Figure S11**	S7
**Figure S12**	S8

UV–vis and fluorescence measurements

The amount of unconjugated dye in the ethanol washing solutions was analyzed as follows: the ethanol filtrate was concentrated to dryness, then, the filtrate was dissolved in DMSO in a volumetric flask and the absorbance (Abs) of the obtained solutions at λ_{max} = 508–514 nm was measured.

Detailed dilution protocol

Washing solutions of **Dex-1a** were diluted as follow using DMSO:

- DMSO solution was firstly diluted to 50 mL, then 1 mL of this solution was diluted to 50 mL.
- The filtrate related to the first washing solution was dissolved in 100 mL.
- The filtrate related to the second washing solution was dissolved in 50 mL.

Washing solutions of **Dex-1b-c** were diluted as follow using DMSO:

- DMSO solution was firstly diluted to 50 mL, then 1 mL of this solution was diluted to 50 mL.
- The filtrate related to the first washing solution was dissolved in 100 mL.
- The filtrate related to the second washing solution was dissolved in 20 mL.

For the dilution curves a note amount of BODIPY-Dex was dissolved in a note amount of water and dilutions were prepared from the stock solution.

Fluorescence spectra were recorded at λ_{exc} = 380 nm or λ_{exc} = 460 nm.

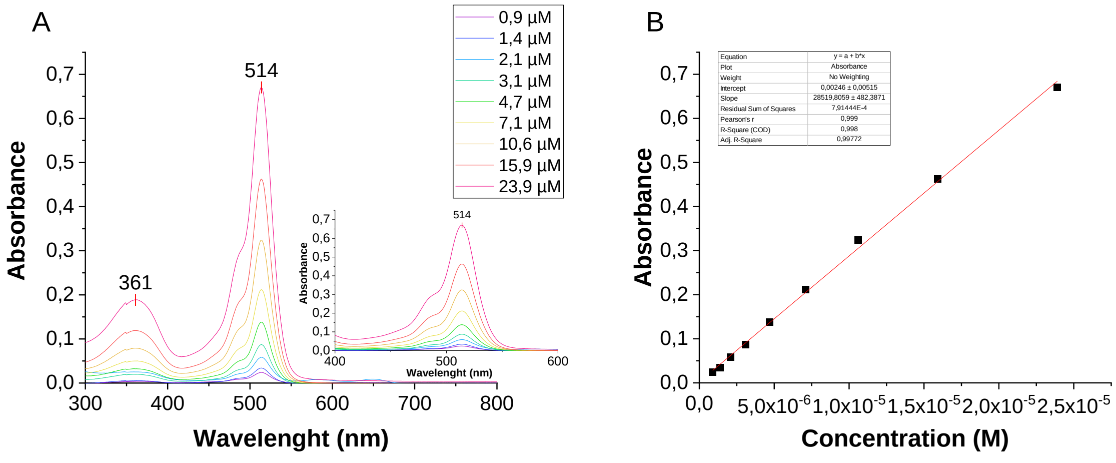

Figure S1: A. UV–vis absorbance spectra of PBA-BODIPY **1** in DMSO; B. Evaluation of the molar extinction coefficient of PBA- BODIPY **1** in DMSO.

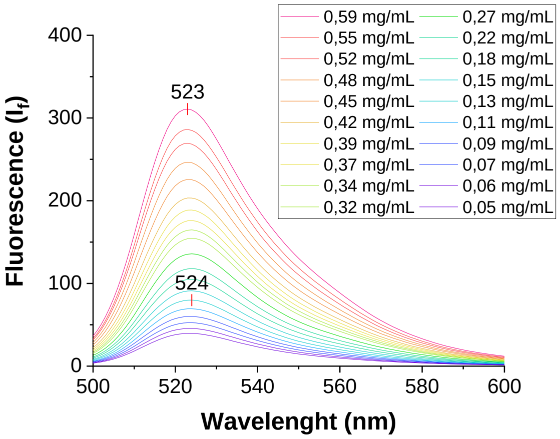

Figure S2: Fluorescence emission spectra of **Dex-1b** (λ_{exc} = 460 nm) in water.

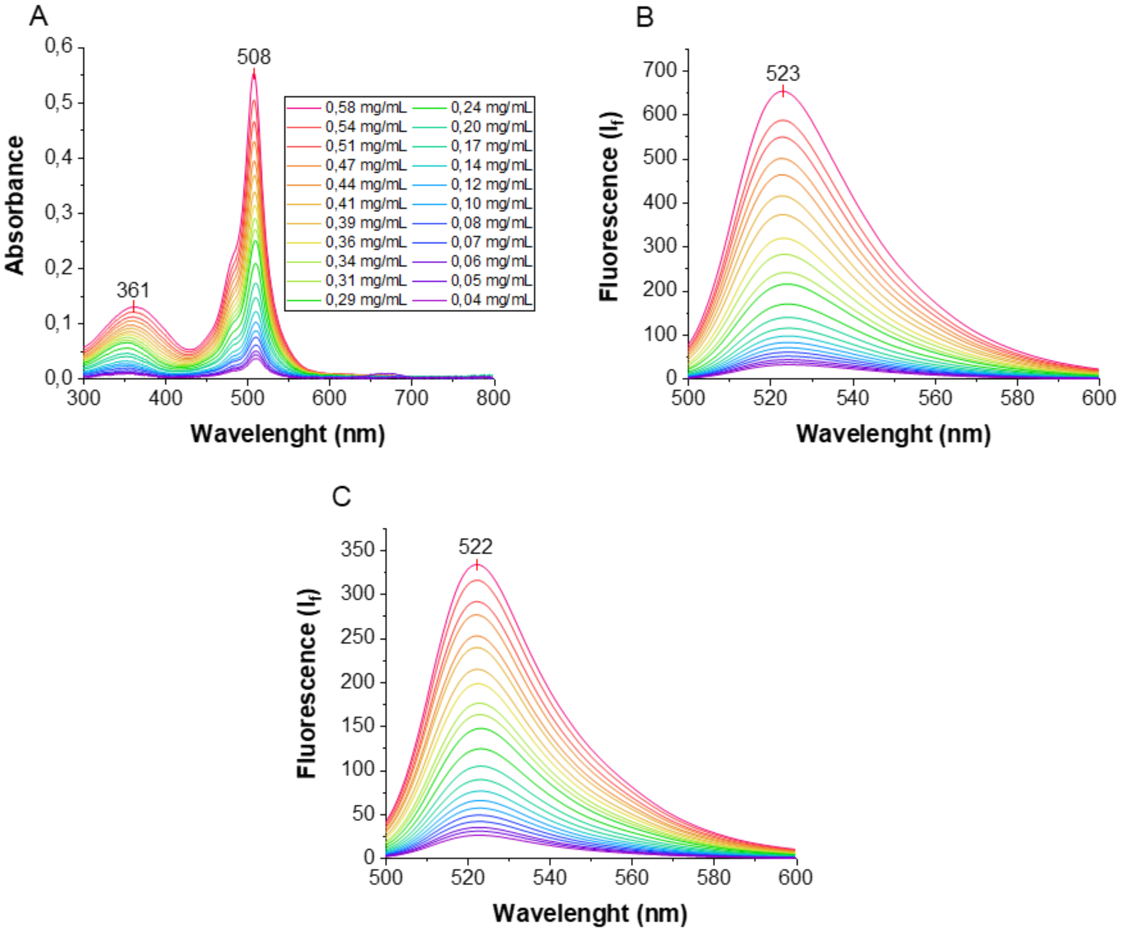

Figure S3: A. UV–vis absorbance spectra and B and C. Fluorescence spectra (λ_{exc} = 380 nm B and λ_{exc} = 460 nm C) of **Dex-1c** solution in water.

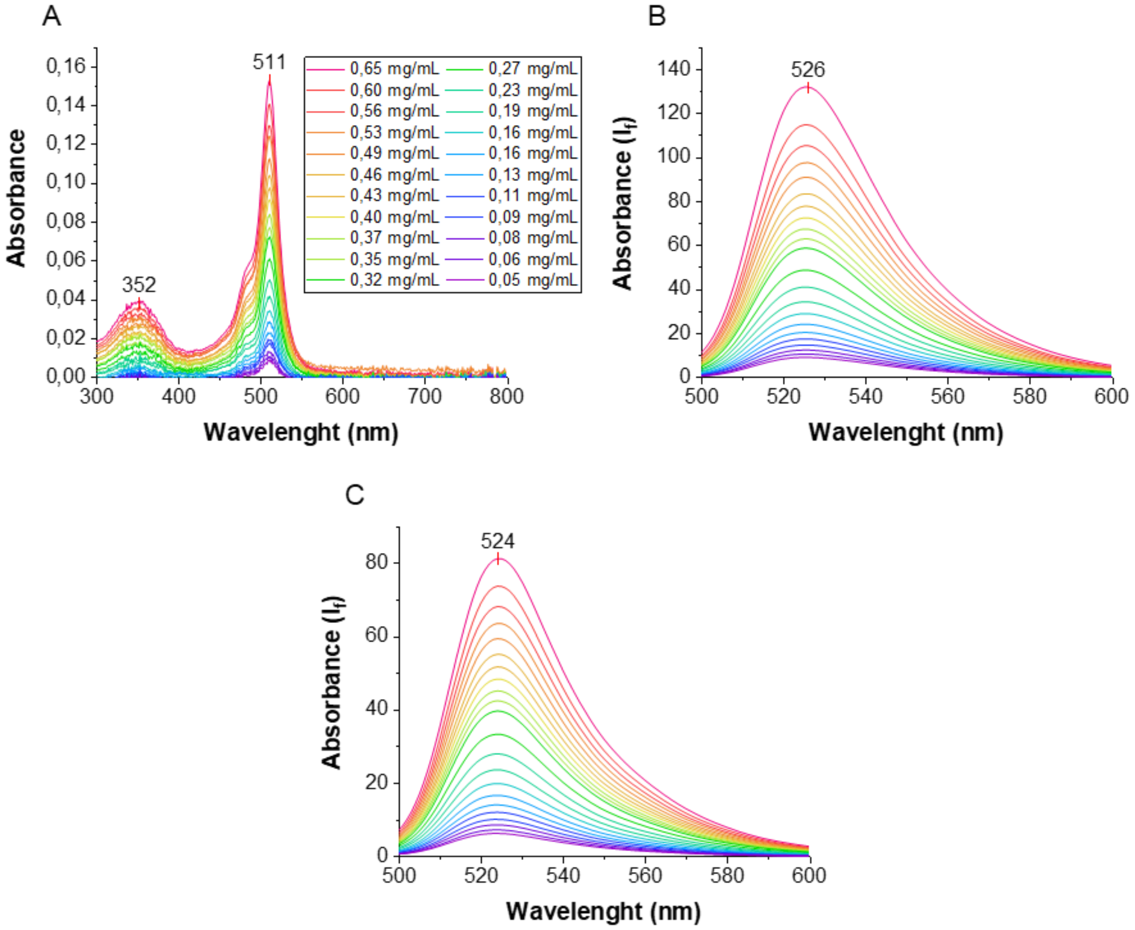

Figure S4: A. UV–vis absorbance spectra and B and C. Fluorescence spectra (λ_{exc} = 380 nm B and λ_{exc} = 460 nm C of **Dex-1d** solution in water.

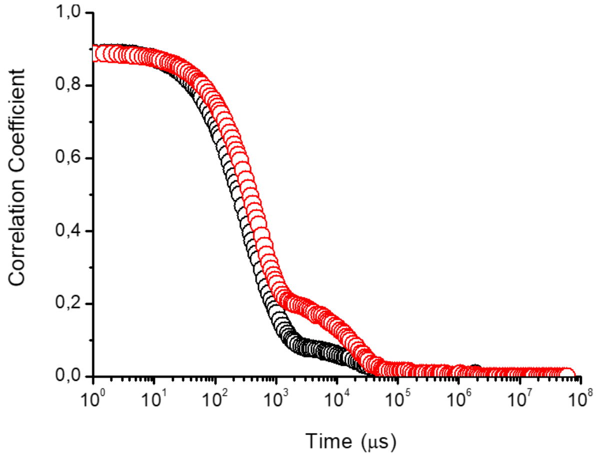

Figure S5: Correlation coefficient by DLS measurements of the conjugate **Dex-1b** (1.0 mg/mL) dispersed in H_2O (black curve) and PBS (red curve).

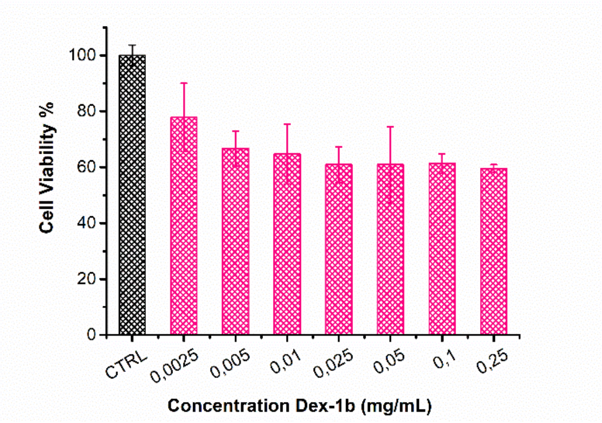

Figure S6: Percentage of cell viability of A549 cells after 24 hours of exposure at various concentrations of the conjugate **Dex-1b** reported as mg/mL concentration of functionalized polymer.

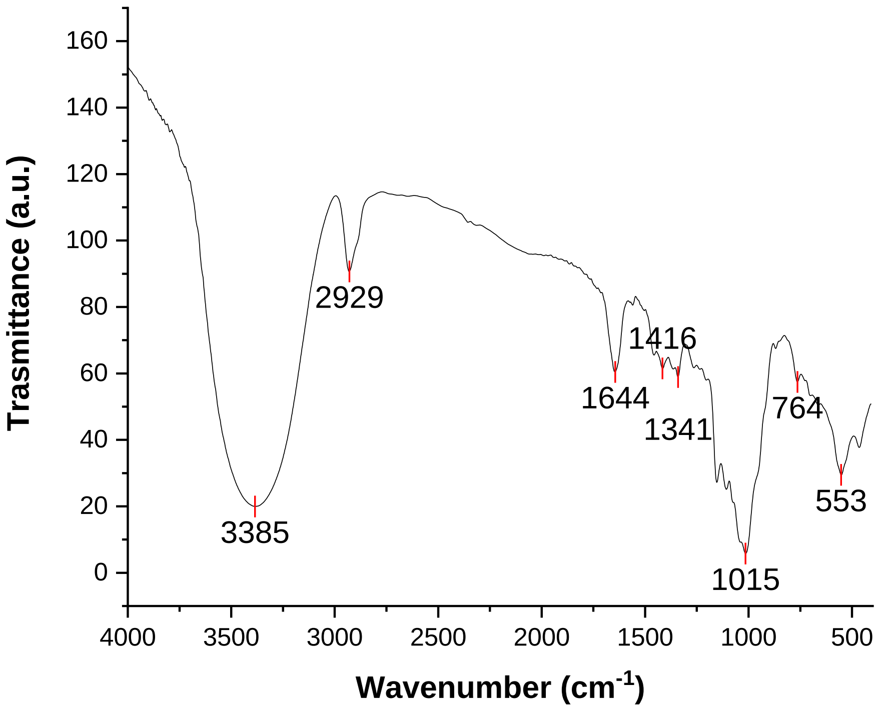

Figure S7: FTIR spectrum of **Dex-1a**.

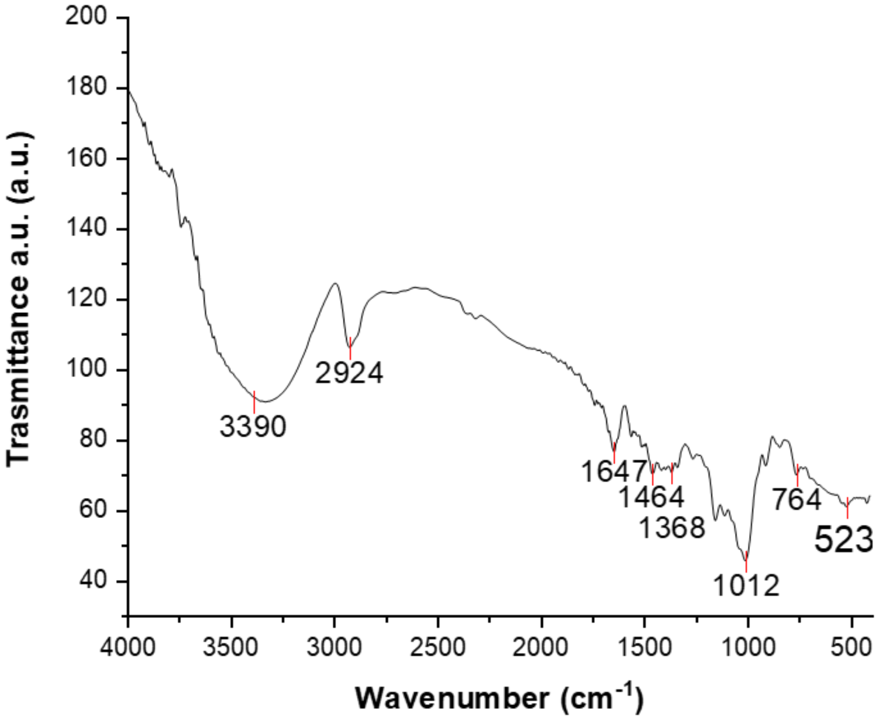

Figure S8: FTIR spectrum **Dex-1b.**

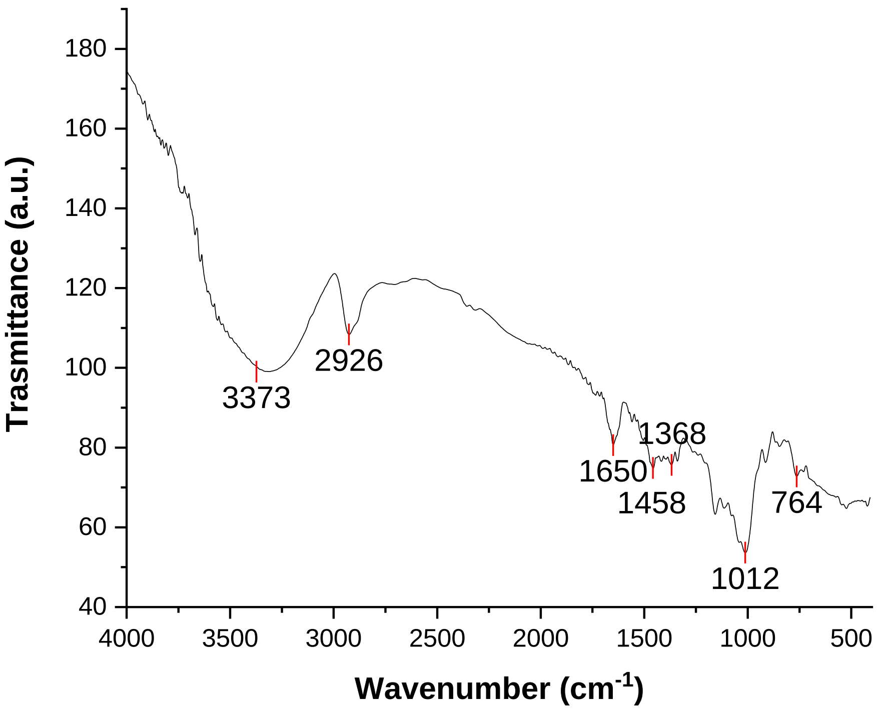

Figure S9: FTIR spectrum of **Dex-1b**.

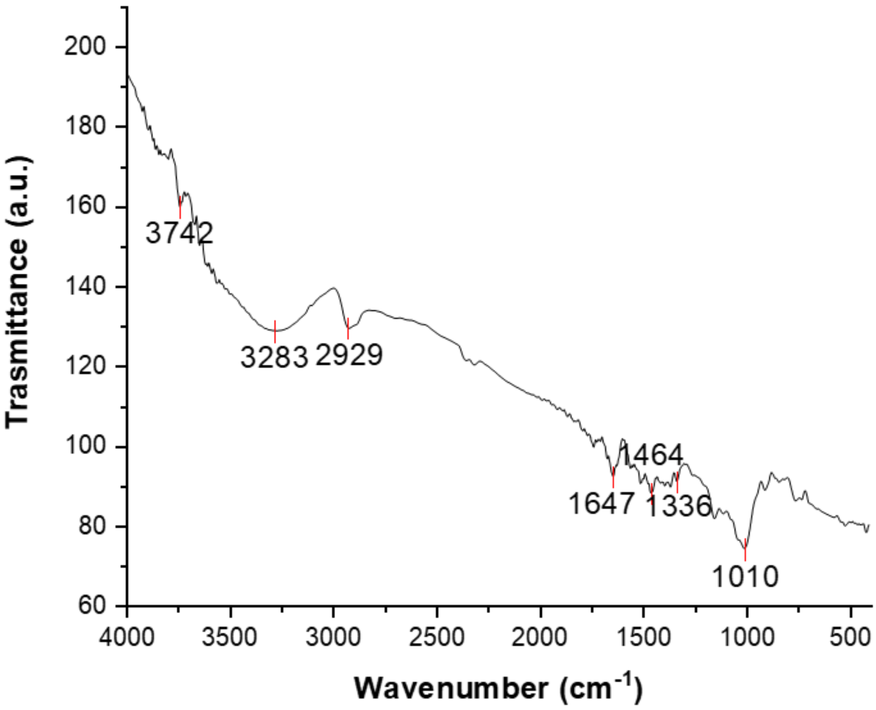

Figure S10: FTIR spectrum of **Dex-1d**.

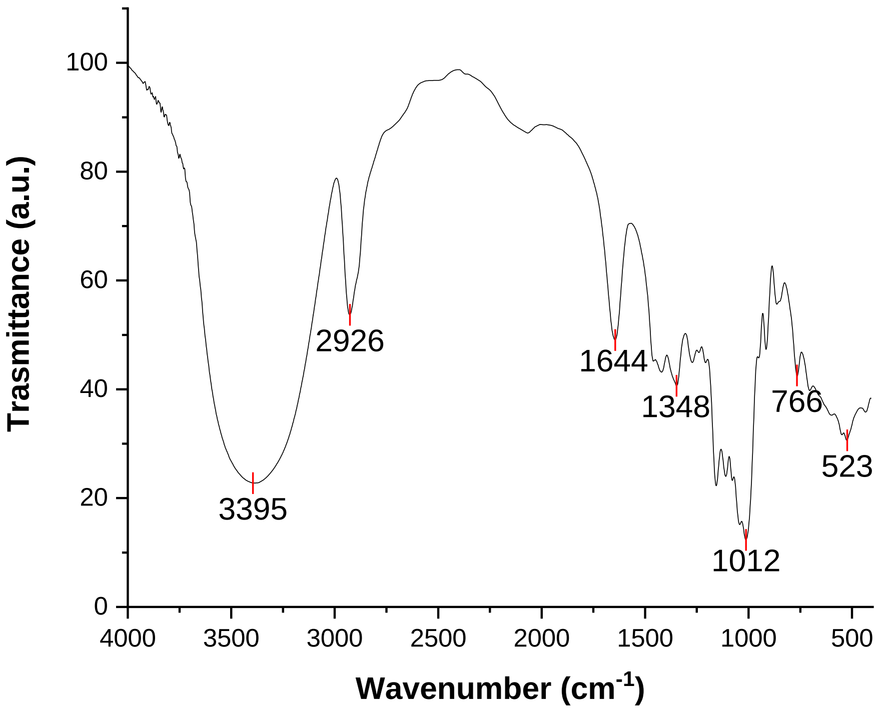

Figure S11: FTIR spectrum of dextran.

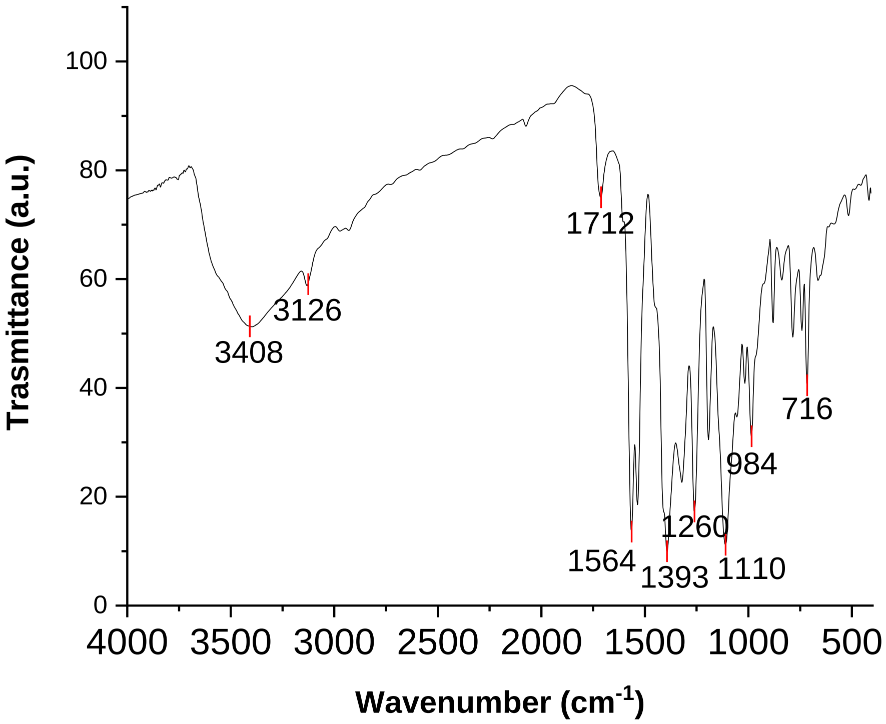

Figure S12: FTIR PBA-BODIPY **1**.
